# Case Report: Endoscopic manifestations and clinical features of small intestinal lymphangioma—A report of two cases

**DOI:** 10.3389/fonc.2022.916295

**Published:** 2022-12-08

**Authors:** Limei Wang, Huang Feng, Bingxin Chen, Fujuan Luan

**Affiliations:** Department of Digestive Diseases, the First Affiliated Hospital of Soochow University, Suzhou, Jiangsu, China

**Keywords:** case report, small intestinal lymphangioma, surgical treatment, clinical features, capsule endoscopy, enteroscopy

## Abstract

**Objective:**

The aims of this study were to analyze the clinical characteristics, auxiliary examinations, and treatment measures of small intestinal lymphangioma and to improve the clinical diagnostic ability of clinicians.

**Methods:**

This paper reports two cases of small intestinal lymphangioma in the Department of Gastroenterology, the First Affiliated Hospital of Soochow University, and makes a comprehensive analysis.

**Results:**

A 31-year-old woman went to the hospital with complaints of dizziness, fatigue, and anemia. A 52-year-old woman complained of upper abdominal pain and went to the hospital with abdominal pain awaiting investigation. Both patients were subjected to three major routine examinations, tumor complete set, CT, capsule endoscopy, and deep enteroscopy, and both of them underwent complete resection of the affected intestinal segment. Pathology showed that both patients had small intestinal lymphangioma.

**Conclusions:**

The clinical manifestations of small intestinal lymphangioma lack specificity. Capsule endoscopy and deep enteroscopy are helpful for clinical diagnosis, and pathological examination is still the gold standard. Surgical treatment can achieve better results.

## Introduction

Small intestinal lymphangioma is a rare disease of the digestive tract. It often presents as chronic gastrointestinal bleeding due to mucosal compression, bleeding, necrosis, or ulceration caused by enlarging the tumor. It accounts for 6% of small intestinal tumors in children and 1.4%–2.4% in adults (1). The clinical symptoms of small intestinal lymphangioma are insidious. It is not easy to detect at an early stage and is often found during physical examination. A study found that abdominal pain, gastrointestinal bleeding, and anemia are the most common clinical symptoms of small intestinal lymphangioma (2). In this paper, anemia and abdominal pain were the main reasons for the admission of two patients. Both patients underwent laparoscopic surgery and had favorable prognosis. Therefore, by reporting two cases in our hospital and reviewing domestic and foreign literature, it aims to provide some help for the diagnosis and treatment of small intestinal lymphangioma in clinical work.

## Case reports

**Figure 1:**
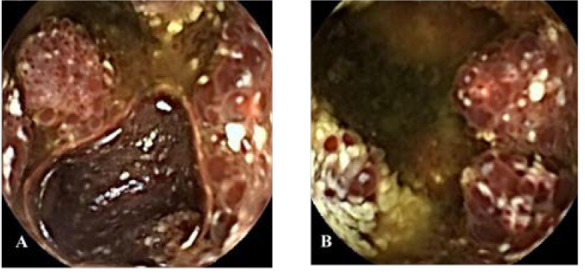
Capsule endoscopy **(A, B)** images of patient A. **(A, B)** Mucosa of the proximal jejunum showed polypoid elevation, surface hyperemia, edema, erosion, white moss attachment, and active bleeding.

**Figure 2:**
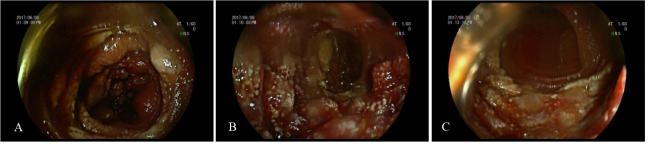
Deep enteroscopy **(A–C)** images of patient A. **(A–C)** Granular hyperplasia of the mucosa 190–200 cm away from the ligament of Treitz, and the surface was erosive, with white speckles attached.

**Figure 3:**
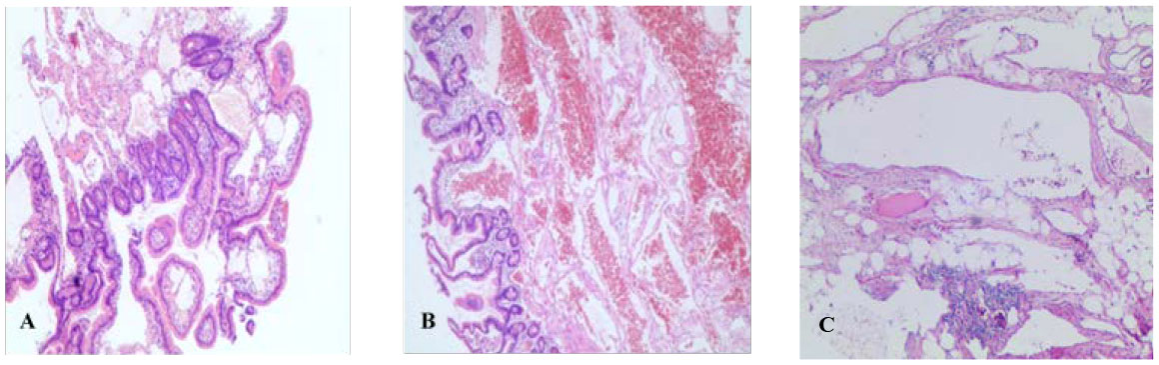
Pathological results (HE staining, ×100). **(A)** Pathology of patient A after deep enteroscopy. **(B)** Postoperative pathology of patient A. **(C)** Postoperative pathology of patient B.

Patient A, a 31-year-old woman, went to the hospital on 1 June 2017 because of “fatigue and dizziness for 1 month and aggravation for 1 week”. The patient developed dizziness and fatigue without obvious inducement 1 month ago, and her symptoms became worse in the past week. Then, she went to the emergency department of our hospital. Blood routine examination showed a hemoglobin (HGB) of 38 g/L, and esophagogastroduodenoscopy showed chronic superficial gastritis. Reexamination after blood replenishment treatment still showed severe anemia, and the symptoms of dizziness and fatigue did not improve significantly. Therefore, the patient was in the hospital due to “anemia of unknown origin”. During hospitalization, the patient did not have nausea, vomiting, abdominal pain, abdominal distension, black stool, and bloody stool. Physical examination showed anemic appearance, soft abdomen, no tenderness, rebound pain, no obvious mass, and bowel sounds six times per minute. Admission diagnosis was as follows: anemia of unknown origin. Blood routine showed HGB 45 g/L; urine routine revealed 94 RBC/μl; stool occult blood test was positive; tumor full set showed erythropoietin >762.00 mIU/ml. Contrast-enhanced CT of chest, abdomen, and pelvis revealed a few fibrosis foci in the left lower lung, small hepatic cysts, and slightly reduced density of heart cavity, consistent with anemia. Capsule endoscopy showed that the mucosa at the proximal end of the jejunum is polypoid, with congestion, edema, erosion, white moss attachment, and active bleeding, as shown in **Figure 1**. Deep enteroscopy showed a granular hyperplasia of the mucosa 190–200 cm away from the ligament of Treitz. The surface was erosive, with white speckles attached, and the distal mucosa was normal, as shown in **Figure 2**. The initial diagnosis of the patient was jejunal lesion (nature unknown because of the patient’s refusal of pathological examination). Under deep enteroscopy, the physician took three pieces of jejunal tissue 190–200 cm away from the ligament of Treitz with a total diameter of 0.2 cm. Pathology revealed chronic inflammation of jejunal mucosa and cystic dilatation of lymphatic vessels in the mucosa propria, as shown in **Figure 3**. The patient received surgical treatment on 14 June 2017. During the operation, the surgeon removed a section of intestine with a length of 17 cm and a diameter of 2–2.5 cm. There is a granular protrusion 5 cm away from the cutting end of one side, with a range of 7 cm × 5 cm × 1.5 cm. At the same time, the cut surface was bleeding, and milky white liquid flowed out from the peripheral adipose tissue. Pathology showed small intestinal angioma, and no lesions involved the resection end, as shown in **Figure 3**. The patient left the hospital after anti-inflammatory and nutritional support treatment.

**Figure 4:**
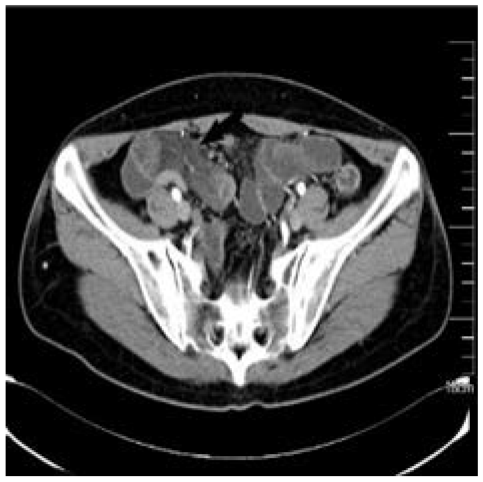
CT of patient B’s abdomen. Pelvic local intestinal wall edema, disorder, and mesenteric edema.

**Figure 5:**
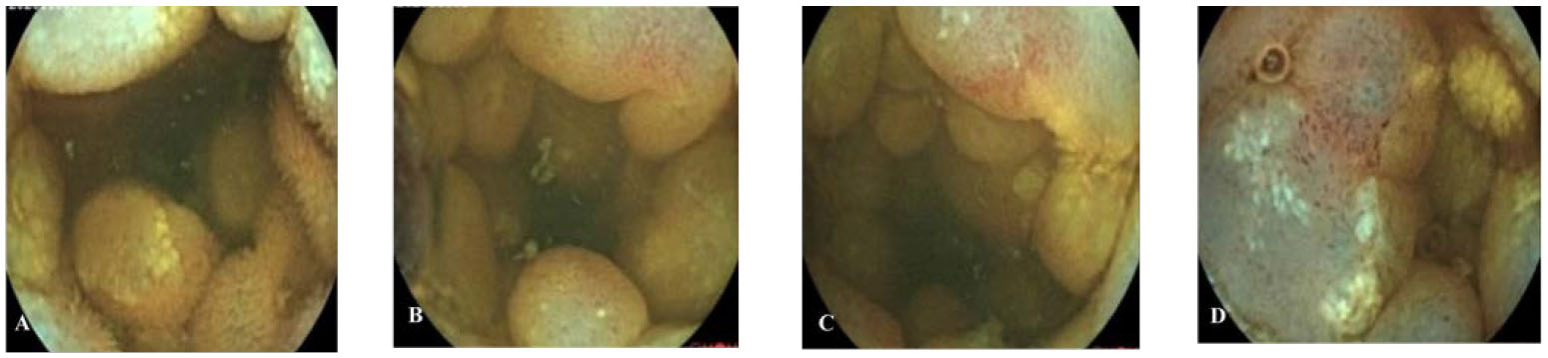
Capsule endoscopy of patient B. **(A–D)** The lesion mucosa showed continuous multiple nodular protrusions, with redness and erosion on the surface.

**Figure 6:**
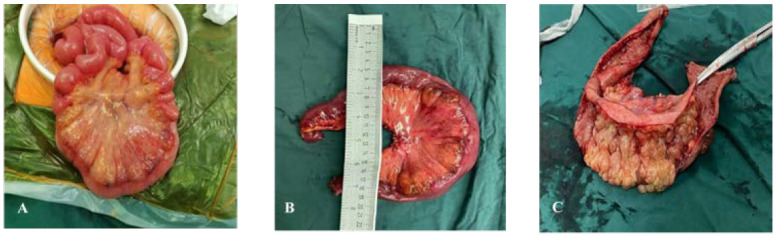
Surgical excision of gross specimen for patient B. **(A)** Diseased bowel piece; **(B)** cut a section of bowel intraoperatively, with a length of 38 cm and a diameter of 4–7 cm; **(C)** 6.5 cm from the end of one incision and 15 cm from the end of the other, a mucous vesicular uplift area of 11 cm × 7 cm can be seen. The mesentery surface is hard in texture, and the section is spongy and gray yellow.

Patient B, a 52-year-old woman, went to the hospital on 3 November 2020 because of “recurrent upper abdominal pain for more than 20 days”. The patient had abdominal pain more than 20 days ago. The pain was mainly in the middle and upper abdomen, which was like a paroxysmal knife cutting and relieved after several seconds. There was no radiating pain in the waist and back, no abdominal distension, and no nausea and vomiting. Physical examination showed tenderness in the middle and right upper abdomen without rebound pain and muscle tension. The abdomen is flat without gastrointestinal type or peristalsis wave, there was no obvious mass, and the bowel sounds are normal. Admission diagnosis revealed abdominal pain of unknown origin. There were no abnormalities in blood routine, CRP, biochemistry, AFP, CA19-9, and CEA. Abdominal enhanced CT showed local intestinal wall edema, structural disorder, and mesenteric edema, as shown in **Figure 4**. Esophagogastroduodenoscopy revealed bile retention fluid in the stomach. Capsule endoscopy showed ileal lesions, with multiple continuous nodular changes in the mucous membrane, and the surface was red and erosive, as shown in **Figure 5**. Contrast-enhanced CT of the small intestine showed segmental intestinal wall thickening in the terminal ileum with obvious edema of the surrounding mesentery, which suggested the possibility of inflammatory bowel disease (IBD). CT three-dimensional reconstruction of small intestine showed segmental intestinal wall thickening in the distal ileum with obvious edema in the surrounding mesangium. After consultation with the imaging department, the patient was suspected to have Crohn’s disease (CD) due to the ileal lesions. However, the clinical features and course of the patient did not conform to the typical manifestations of CD, so surgical treatment was important for diagnosis and treatment. The patient underwent surgical treatment on 1 December 2020. Intraoperative exploration revealed a 30-cm-long part of the small intestine with obvious edema and thickening 4.5 m away from the ligament of Treitz. The intestinal lumen was narrow and the mesangial surface was uneven, showing cystic jelly-like changes. During operation, the operator dissected a piece of intestine with a length of 38 cm and a diameter of 4–7 cm. There is an 11 cm × 7 cm mucous bulbous area 6.5 cm away from the cut end on one side and 15 cm from the cut end on the other side. The mesenteric surface was hard in texture, and the cut surface was spongy and gray yellow, as shown in **Figure 6**. Pathology indicated small intestinal lymphangioma, and no cancer involved both ends, as shown in **Figure 3**. After the operation, she left the hospital after receiving anti-inflammatory and nutritional support therapy.

## Discussion

Lymphangioma is a benign tumor caused by congenital malformation of the lymphatic system (3). It usually occurs in the head, neck, and axilla of children. Lymphangioma occurring in the abdominal cavity is rare, especially in adults. The most common site of small intestinal lymphangioma was the mesentery, followed by omentum, retromesentery, and retroperitoneum (4). Mesenteric lymphoma is rare, accounting for less than 1% of all lymphangiomas (5, 6).

Through literature review, we found that there are no characteristic clinical manifestations in the early stage of small intestinal lymphangioma. With the enlargement of the tumor, it could cause various clinical symptoms. The common symptoms were abdominal pain caused by intestinal irritation, followed by gastrointestinal bleeding and anemia, intestinal obstruction, and hypoproteinemia. Some cases presented with non-specific gastrointestinal symptoms, such as nausea, vomiting, abdominal distension, diarrhea, and anorexia (7–9). In the process of clinical diagnosis and treatment, doctors rely solely on clinical manifestations, which could easily result in missed diagnosis and misdiagnosis. Extra examination can help to further clarify the diagnosis. Imaging examination has suggestive significance, among which CT examination has high sensitivity. It has more advantages among many imaging examination methods and is the first choice for patients with suspected small intestinal lymphangioma (2, 10). CT showed clear, non-enhanced cystic lesions (2). In gastrointestinal endoscopy, capsule endoscopy and deep enteroscopy play an important role in the diagnosis of small intestinal diseases. Capsule endoscopy can help locate small intestinal masses by directly viewing the whole small intestinal mucosa, which is one of the preferred methods for diagnosing small intestinal diseases (11). Microscopically, small intestinal lymphangioma showed multiple red grape-like nodules, vegetable patterns, or polypoid lesions on the intestinal wall, with erosion, ulceration, and bleeding on the surface. Deep enteroscopy can detect lesions and biopsy for pathological examination, which has a higher diagnostic value than other endoscopic methods (12). In recent years, capsule endoscopy and deep enteroscopy has improved the detection rate of small intestinal lymphangioma. However, since the lesion is located in the small intestine, endoscopic examinations often fail to detect the lesion site and thus miss the diagnosis. Therefore, for most patients, a diagnosis was made after surgical resection of the lesion and submission for pathology (13). Histopathological examination is still the gold standard for diagnosis. A large lymphatic cavity can be seen from the specimen under the microscope, with collagen and smooth muscle covered on the surface. Although small intestinal lymphangioma is a benign tumor, it can still show borderline changes and there is a risk of malignant transformation into lymphangiosarcoma. At the same time, we recommend patients to undergo radical surgical resection because the lesions may further grow and compress adjacent organs, resulting in volvulus, intestinal obstruction, secondary infection, rupture, bleeding, and other complications (14).

Small intestinal lymphangioma should be distinguished from intestinal endometriosis. Endometriosis refers to the growth of endometrial tissue outside the uterine cavity and myometrium. When ectopic endometrium invades the intestine, it is intestinal endometriosis. The disease has no specific clinical manifestations. When it invades the rectum and sigmoid colon, a series of intestinal symptoms such as abdominal pain, diarrhea, constipation, hematochezia, and even intestinal obstruction can occur. It is difficult to distinguish the two diseases based on clinical manifestations, and thus, it could easily result in misdiagnosis. The common diagnostic methods of this disease include ultrasound, CT, colonoscopy, and laparoscopy. Ultrasonography mainly showed thickening of the affected intestinal wall and hypoechoic changes (15, 16). Colonoscopy may show thickening of the intestinal wall at the lesion site, polypoid changes in the mucosa, and local eminence or intestinal lumen stenosis (17). Pathological examination is the best examination method. However, due to the lack of characteristic microscopic manifestations in the early stage of this disease and the lack of recognition of the characteristics of this disease by clinicians, the detection rate of endoscopic biopsy for pathological examination is low. Therefore, surgical treatment is the first choice for symptomatic patients with intestinal endometriosis, and pathological examination of the specimens removed during surgery can greatly improve the detection rate of the disease and achieve the purpose of treatment.

In this paper, two patients went to the hospital with anemia and abdominal pain as the main symptoms. CT examination of patient A showed no abnormality, while patient B showed intestinal wall edema and disorder; thus, a diagnosis could not be made. Capsule endoscopy and deep enteroscopy also failed to diagnose small intestinal lymphangioma. As patient B showed symptoms of IBD, other intestinal manifestations may cover up lymphangioma-related symptoms, leading to a missed diagnosis. The diagnosis is clear by pathological results, which further proved that pathological examination is still the gold standard for the diagnosis of small intestinal lymphangioma. According to the size and shape of the proliferative lymphatic vessels in the tumor, lymphangioma can be classified into three types: capillary lymphangioma, cavernous lymphangioma, and cystic lymphangioma (5). Sometimes, the three types of lymphatic vessels can coexist, which is difficult to classify. Surgical treatment is the gold standard treatment for small intestinal lymphangioma. In this study, both patients underwent laparoscopic exploratory laparotomy combined with partial small bowel resection to remove the diseased intestinal segment. Intraoperative pathology was performed on the diseased intestinal segment during the operation. Both patients recovered well after anti-inflammatory and nutritional support treatment, and the disease has not recurred so far.

In conclusion, small intestinal lymphangioma is a rare tumor of the small intestine with a lack of clinical specificity. Imaging examination is of certain significance, and capsule endoscopy and deep enteroscopy are helpful for clinical diagnosis. The pathological results can make a definite diagnosis of small intestinal lymphangioma. Small intestinal lymphangioma is a benign tumor, but malignant transformation may exist. Surgical treatment is the first choice, with the advantages of quick recovery and good prognosis.

## Data availability statement

The original contributions presented in the study are included in the article/Supplementary Material. Further inquiries can be directed to the corresponding author.

## Ethics statement

The studies involving human participants were reviewed and approved by Ethics Committee of the First Affiliated Hospital of Soochow University. The patients/participants provided their written informed consent to participate in this study. Written informed consent was obtained from the individual(s) for the publication of any potentially identifiable images or data included in this article.

## Author contributions

FL contributed to conception and design of the study. LW wrote the first draft of the manuscript. HF and BC wrote sections of the manuscript. All authors contributed to manuscript revision, read, and approved the submitted version.
